# A Novel Vector-Based Method for Exclusive Overexpression of Star-Form MicroRNAs

**DOI:** 10.1371/journal.pone.0041504

**Published:** 2012-07-19

**Authors:** Bo Qu, Xiao Han, Yuanjia Tang, Nan Shen

**Affiliations:** 1 Joint Molecular Rheumatology Laboratory of the Institute of Health Sciences and Shanghai Renji Hospital, Shanghai Institutes for Biological Sciences, Chinese Academy of Sciences, and Shanghai Jiaotong University School of Medicine, Shanghai, People's Republic of China; 2 Key Laboratory of Stem Cell Biology, Shanghai Institutes for Biological Sciences, Chinese Academy of Sciences, Shanghai, People's Republic of China; 3 Division of Rheumatology and the Center for Autoimmune Genomics and Etiology (CAGE), Cincinnati Children's Hospital Medical Center, Cincinnati, Ohio, United States of America; University of Barcelona, Spain

## Abstract

The roles of microRNAs (miRNAs) as important regulators of gene expression have been studied intensively. Although most of these investigations have involved the highly expressed form of the two mature miRNA species, increasing evidence points to essential roles for star-form microRNAs (miRNA*), which are usually expressed at much lower levels. Owing to the nature of miRNA biogenesis, it is challenging to use plasmids containing miRNA coding sequences for gain-of-function experiments concerning the roles of microRNA* species. Synthetic microRNA mimics could introduce specific miRNA* species into cells, but this transient overexpression system has many shortcomings. Here, we report that specific miRNA* species can be overexpressed by introducing artificially designed stem-loop sequences into short hairpin RNA (shRNA) overexpression vectors. By our prototypic plasmid, designed to overexpress hsa-miR-146b-3p, we successfully expressed high levels of hsa-miR-146b-3p without detectable change of hsa-miR-146b-5p. Functional analysis involving luciferase reporter assays showed that, like natural miRNAs, the overexpressed hsa-miR-146b-3p inhibited target gene expression by 3′UTR seed pairing. Our demonstration that this method could overexpress two other miRNAs suggests that the approach should be broadly applicable. Our novel strategy opens the way for exclusively stable overexpression of miRNA* species and analyzing their unique functions both *in vitro* and *in vivo*.

## Introduction

MicroRNAs (miRNAs) are endogenous noncoding RNAs, which are ∼22 nucleotides (nt) long and play important regulatory roles in many biological functions in plants and animals [1]. MiRNAs can influence the expression levels of many proteins mainly by targeting mRNAs for degradation or translational repression [2], [3]. Besides, many new findings, such as miRNAs could promote mRNA translation [4], [5] or even participate in the regulation of mRNA transcription [6], [7], were added to the complexity of the mechanisms which were used by miRNAs to shape gene expression. Thus, miRNAs are involved in a wide range of physiological and pathological processes [8], [9], [10].

The genomic DNA sequences that encode miRNAs are located mostly in introns or intergenic regions [11]. After transcription by RNA polymerase II (RNA Pol II), the first step in the biogenesis of miRNA involves the cleavage of the primary miRNA transcript (pri-miRNA) by Drosha in the nucleus to produce a ∼60–100 nt hairpin-like structure called the precursor miRNA (pre-miRNA). The pre-miRNA is subsequently transported out of the nucleus by Exportin-5, where it is further processed by Dicer to generate a ∼22 nt duplex RNA product. Finally, one strand of the duplex is incorporated into the Argonaut-containing RNA-induced silencing complex (RISC) [12]. Incorporation into RISC is a biased process that one strand of the duplex is preferred to be associated with RISC and accumulates to a higher steady-state level than the other strand which is variously called either the passenger strand or the miRNA* species [13], [14] (Note that: If there were not enough data to determine which one was expressed predominantly, the mature sequences would be assigned names by adding −5p (from the 5′ arm) and −3p (from the 3′ arm) to the end. So, the miRNA* species could be originally named −5p or −3p) [15].

Since the discovery that miRNAs are found in many species and expressed ubiquitously in nearly all tissues and organs [1], [16], considerable effort has been devoted to explore the function of miRNAs during health and disease. However, most investigations have focused on the more highly expressed of the two mature sequences and less attention has been paid to the passenger strand and it was thought to be degraded soon after processing. Thus, the potential regulatory activity of miRNA* species has been comparatively underestimated. Nonetheless, several studies have demonstrated the existence of miRNA* species in different organisms [17], [18], [19], [20], [21], [22], [23], and accumulated to substantial levels in certain tissues under physiological conditions or in some cell type with specific stimulation [24], [25], [26]. Indeed, certain miRNA* species associate with RISC [27], [28], function like their highly expressed counterparts [26], [29], and are implicated in the pathogenesis of some diseases or in regulating signaling events [24], [30], [31].

Gain-of function experiments are frequently used to explore the regulatory functions of miRNAs. Until now, miRNAs have been overexpressed either by transfecting cells with synthetic miRNA mimics or by introducing miRNA expression plasmids into cells [32]. Synthetic miRNA mimics have the ability to specifically elevate levels of miRNA* species. Nonetheless, given that their use only permits transient expression, they cannot be used for long term studies or *in vivo* experiments [32]. Commercially available mimics are usually expensive and often designed to favor the expression of the miRNA strand by manipulating the sequence of the miRNA* strand [29]. This makes them unsuitable for investigations concerning the function of miRNA* species. Vector-based miRNA expression systems can be used to ensure stable expression of miRNAs. However, the biased nature of strand incorporation into RISC makes it challenging to express high levels of the miRNA* strand while simultaneously eliminating the side effects caused by the miRNA strand [29].

To address this challenge and provide an easy and inexpensive way to express specific miRNA* species, we reasoned the possibility to overexpress specific miRNA species by using manipulated short hairpin RNA (shRNA) overexpression system and developed a new expression strategy. Further tests on our prototypic plasmid, plvx-hs-146b-3p, which was designed to overexpress hsa-miR-146b-3p for our further functional studies, showed that our plasmid can express high levels of the miRNA* species (hsa-miR-146b-3p) with no alteration of the level of endogenous hsa-miR-146b-5p miRNA. And the expressed hsa-miR-146b-3p could function to repress gene expression through 3′UTR binding. Our strategy provides new opportunities to analyze the functions of miRNA* species *in vitro* and *in vivo*.

## Materials and Methods

### Cell culture and reagents

HeLa cells (provided by Youcun Qian, the Institute of Health Sciences, Shanghai Institutes for Biological Sciences, Chinese Academy of Sciences, and Shanghai Jiaotong University School of Medicine, Shanghai, China; [33]), HEK 293T cells and Hep G2 cells (obtained from Cell Bank, Shanghai Institutes for Biological Sciences, Chinese Academy of Sciences, Shanghai 200032, China) were maintained in Dulbecco's Modified Eagle's Medium (Gibco, Invitrogen) supplemented with 10% (v/v) fetal bovine serum (SAFC, Sigma), 100 U/mL penicillin, and 100 µg/mL streptomycin (Gibco, Invitrogen) at 37°C in a humidified atmosphere containing 5% CO_2_. MicroRNA mimics and inhibitors were synthesized by Genepharma (China).

### Generation of plasmids

Details concerning the design of miRNA overexpression plasmids are provided in the first part of the Results section. To generate the DNA incorporated into these plasmids, we simply annealed the two specified synthetic DNA primers (Sangon Biotech, China) in an annealing buffer (Beyotime, China) according to the manufacturer's instructions. Briefly, the reaction mixture contained 10 μL of annealing buffer, 20 μL of ddH_2_O, 10 μL of upstream primer (50 μM), and 10 μL of downstream primer (50 μM). The annealing reaction involved heating the mixtures to 95°C for 2 min, and then decreasing the temperature by 1°C every 90 s until a temperature of 25°C was reached. Finally, the inserts were inserted either into plvx-shRNA2 vectors (Clontech) using *Bam*H1 and *Eco*R1 restriction sites for overexpression plasmids, or into psicheck vectors (Promega) using *Not*1 and *Xho*1 restriction sites, or into pMIR-Report vectors (Ambion) using *Mlu*1 and *Sac*1 restriction sites for sensor plasmids. pSIF-ctrl and pSIF-hs-146b plasmids were kindly provided by Mofang Liu's lab at Shanghai Institutes for Biological Sciences, Chinese Academy of Sciences, Shanghai, China. This was approved by the institutional Ethics Review Board of the Institute of Health Sciences (IHS Ethics Review Board), with all subjects providing written informed consent.

The sequences of the DNA primers are listed below.

For plvx-hs-146b-3p:

5′GATCCCCCCAGGATTGAGTCCACAGGGCATTCAAGAGATGCCCTGTGGACTCAGTTCTGGTTTTTA3′

5′AATTTAAAAACCAGGATTGAGTCCACAGGGCATCTCTTGAATGCCCTGTGGACTCAGTTCTGGGGG3′

For plvx-hs-127-3p:

5′GATCCCCAGCCAAGCTCAGACGGATCCGATTCAAGAGA TCGGATCCGTCTGAGCTTGGCTTTTTTA3′

5′AATTTAAAAA AGCCAAGCTCAGACGGATCCGA TCTCTTGAATCGGATCCGTCTGAGCTTGGCTGGG3′

For plvx-hs-142-3p:

5′GATCCCCTCCATAAAGTAGGAAACACTACATTCAAGAGATGTAGTGTTTCCTACTTTATGGATTTTTA3′

5′AATTTAAAAA TCCATAAAGTAGGAAACACTACA TCTCTTGAA TGTAGTGTTTCCTACTTTATGGA GGG3′

For psicheck-hs-146b-3p:

5′TCGACACCCCAGAACTGAGTCCACAGGGCAGGTCAACAATCACCCCAGAACTGAGTCCACAGGGCAGGC3′

5′GGCCGCCTGCCCTGTGGACTCAGTTCTGGGGTGATTGTTGACCTGCCCTGTGGACTCAGTTCTGGGGTG3′

For psicheck-hs-146b-5p:

5′TCGACACCAGCCTATGGAATTCAGTTCTCAGGTCAACAATCACCAGCCTATGGAATTCAGTTCTCAGGC3′

5′GGCCGCCTGAGAACTGAATTCCATAGGCTGGTGATTGTTGACCTGAGAACTGAATTCCATAGGCTGGTG3′

For pMIR-hs-146b-3p:

5′CACCCCAGAACTGACAGCACAGGGCAGGTCAACAATCACCCCAGAACTGACAGCACAGGGCAGGC3′

5′CGCGGCCTGCCCTGTGCTGTCAGTTCTGGGGTGATTGTTGACCTGCCCTGTGCTGTCAGTTCTGGGGTGAGCT3′

For pMIR-hs-146b-3p-mut:

5′CACCCCAGAACTGACAGCAGACGGGAGGTCAACAATCACCCCAGAACTGACAGCAGACGGGAGGC3′

5′CGCGGCCTCCCGTCTGCTGTCAGTTCTGGGGTGATTGTTGACCTCCCGTCTGCTGTCAGTTCTGGGGTGAGCT3′

### Transfection

For luciferase assays, cells were seeded in a 96-well flat-bottomed plate the day before transfection. Seeding was at a concentration of 1×10^4^ cells per well in 100 µL medium containing 10% fetal bovine serum. On the second day, at a confluence of 70%–80%, the cells were transfected with the indicated combination of sensor plasmids and miRNA overexpression vectors or miRNA mimics or inhibitors.

To evaluate miRNA overexpression, the day before transfection, cells were seeded in a 24- well flat-bottomed plate at a concentration of 4×10^4^ cells per well in 500 µL medium containing 10% fetal bovine serum. On the second day, at a confluence of 70%–80%, the cells were transfected with the miRNA overexpression vectors indicated.

Transfection was done using Lipofectamine 2000 (Invitrogen) according to the manufacturer's instructions. We used1 μL of Lipofectamine 2000 for 1 μg of plasmid or 0.5 μL of Lipofectamine 2000 with 10 pmol of miRNA mimics or inhibitors. The final volume for 96-well plates was 100 μL and 600 μL for 24-well plates. After 4 h incubation, the medium was changed to normal culture medium.

### Quantitative real-time RT–PCR

After 24 h of incubation following transfection, total cellular RNA was collected and extracted with TRIzol Reagent (Invitrogen). MiRNA expression was detected using TaqMan MicroRNA Expression Assays (Applied Biosystems) according to the manufacturer's protocol. We used RNU48 as the endogenous reference gene. When indicated, miScript 2 RT kit (QIAGEN) was used to do reverse transcription, and in this case miRNA was detected using SYBR-green based method (SYBR® Premix Ex Taq™ II, TaKaRa). The amplification was performed on ABI PRISM 7900 Real Time PCR System (Applied Biosystems).

### Luciferase assays

Protein was collected 24 h after transfection, using the Passive Lysis Buffer (30 μL per well) provided as part of the Dual-Luciferase Reporter Assay System kit (Promega). Firefly and Renilla luciferase activities were examined according to the procedure outlined by the manufacturer of the Dual-Luciferase Reporter Assay System (Promega). Signal was detected using a Centro XS3 LB960 Luminometer (Berthold).

## Results

### Construction of plasmids for expression of microRNA* species

Researchers have traditionally used plasmids to overexpress miRNA by cloning genomic sequences of the miRNA hairpin precursor into an overexpression vector. These include appropriate stretches of flanking sequences (at least 40 nt on each side) needed for correct processing of the primary transcripts by Drosha [34]. However, because of the biased maturation process of miRNAs, this method merely simulates the endogenous miRNA processing pathway and is unsuitable for either high-level expression of miRNA* species or the specific expression of the miRNA* species without concomitant accumulation of its more highly expressed miRNA counterpart [29].

**Figure 1:**
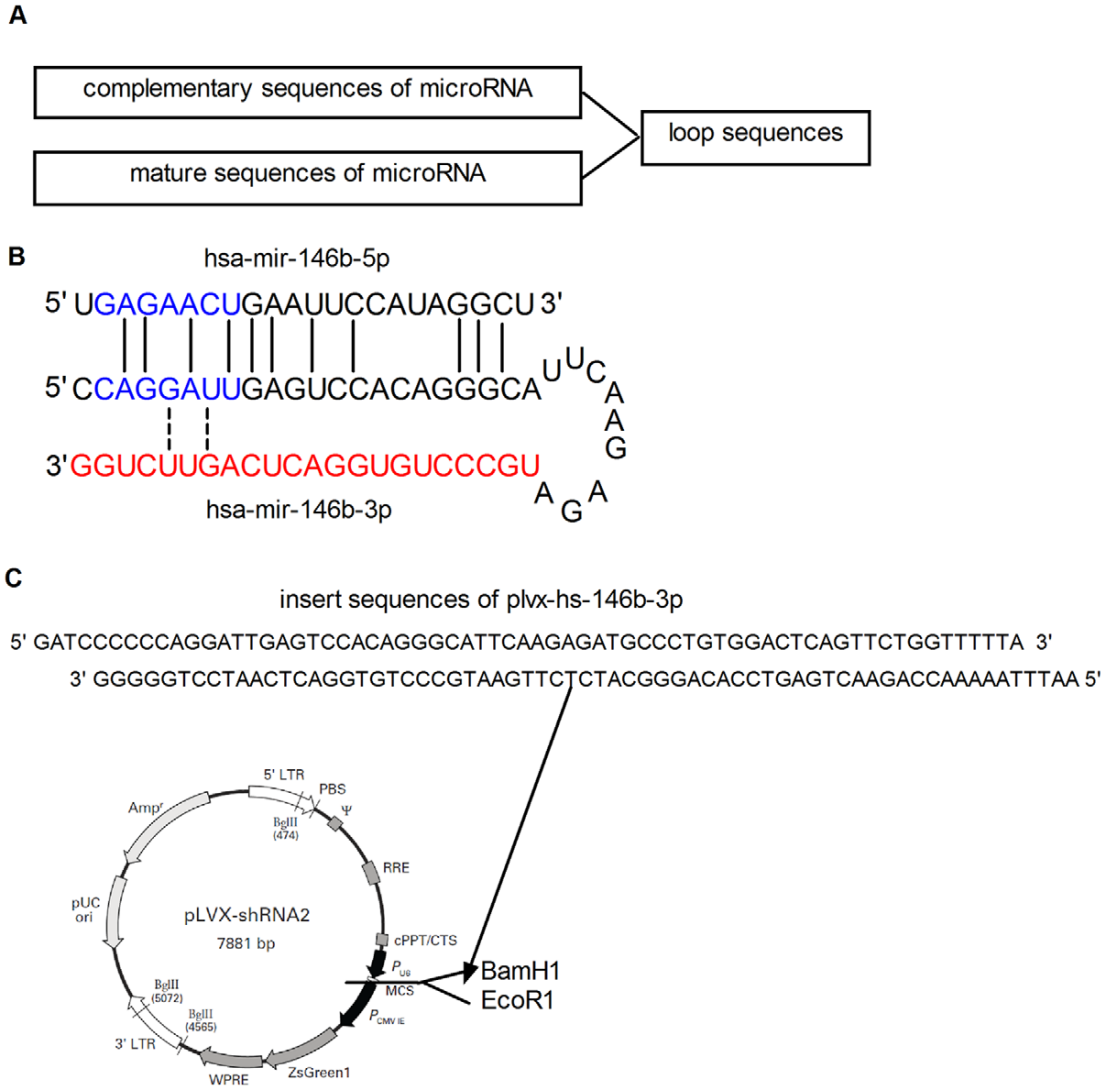
Design and generation of plasmids for the overexpression of microRNA* species. (A) Design of the stem-loop miRNA precursor. The mature sequences of miRNA* species were incorporated into the 3′ arm of the precursor, whereas the complementary sequences of the miRNA* species were incorporated into the 5′ arm. Note that the complementary sequences could either be completely complementary to the miRNA* species or could be modified by mutating several bases in its seed region, which were not complementary to the miRNA* species. (B) The designed hsa-miR-146b-3p stem-loop precursor. The red characters in the 3′ arm correspond with the sequences of hsa-miR-146b-3p. The 5′ arm sequences are the modified complementary sequences of hsa-miR-146b-3p, and the sequences above are hsa-miR-146b-5p, with blue characters denoting seed sequences and solid lines between them indicating the similarity of the two sequences. The dotted lines show the modified sites in the 5′ arm sequences and their corresponding nucleotides in hsa-miR-146b-3p sequences. (C) The uppermost oligo is the DNA insert of hsa-miR-146b-3p precursor obtained by annealing two single stranded DNA oligos with *Bam*H1 and *Eco*R1 sticky ends. The DNA insert was incorporated into plvx-shRNA2 between the recognition sites for the restriction enzymes *Bam*H1 and *Eco*R1. The representation of the vector was modified from an illustration in the instructions provided with the product.

Based on the widely used method for overexpressing miRNAs by transfecting synthetic miRNA mimics, we assumed that if we could introduce exogenous double stranded RNAs containing mature sequences of miRNA* species into cells, we should be able to overexpress the functional miRNA* species. Further, considering that a vector-based shRNA-directed RNA interference system can synthesize customized double stranded short interfering RNAs (siRNAs) in transfected cells [35], [36], [37], we designed an artificial miRNA hairpin precursor (Figure 1a). This involves placing the mature miRNA* sequence in the 3′ strand of the stem, its complementary sequence in the other strand, and a loop sequence between the two. The corresponding DNA template is then cloned into the shRNA overexpression vector.

Several core factors and mechanisms are common between the processes required for the biogenesis and function of both siRNAs and miRNAs [38]. Therefore, we reasoned that the hairpin precursor that mimics shRNAs used for synthesizing siRNAs should give rise to intermediate double-stranded RNAs containing the mature miRNA* sequences that could be incorporated into the RISC complex and function in the same way as miRNAs. Unlike the original miRNA precursors that usually contain mismatches and internal loops in the stems of their hairpin structures [39], [40], the 5′ strand of the designed hairpin precursor was designed to possess complete complementarity with the miRNA* sequences. Although the 5′ strand still contained considerable similarities to the sequences of the miRNA originated from the same precursor of the miRNA*, the change of the order of the sequence and the additional nucleotides made the 5′ strand a different miRNA like RNAs harboring new seed sequences and having new supplementary pairing abilities. In addition, we could modify the 5′ strand by mutating several nucleotides both to further disturb the sequences located in the seed region and avoid seed pairing by these sequences, which might mimic the activity of the “miRNA” species. Owing to the well-established role of seed pairing in miRNA target recognition [41], the modified 5′ strand would not contribute side effects associated with its activity as the “miRNA” strand, thereby enabling specific expression of miRNA* species with no interference from its counterpart miRNA.

To validate our strategy and for our own further functional studies, we focused on hsa-miR-146b-3p, the expression of which is enhanced upon exposure to particular stimuli (data unpublished). We used plvx-shRNA2 as the overexpression vector to make our prototype overexpression plasmid referred as plvx-hs-146b-3p, because this vector could be used to produce lenti-virus particle for stable transfection and *in vivo* application. Hsa-miR-146b-3p was the miRNA* species produced from the hsa-miR-146b precursor, which also gave rise to the more abundant hsa-miR-146b-5p miRNA species. We first designed the hairpin precursor for hsa-miR-146b-3p by including all of its complete complementary sequences (referred as anti-146b-3p) in the 5′ arm and its own sequences in the 3′ arm of the hairpin, and separating them by insertion of a widely used 9-nt loop sequence (UUCAAGAGA) [36]. The same six nucleotides are found in the seed region (site 3–8) of both hsa-miR-146b-5p and anti-146b-3p. We thus changed A to G at site 5 and C to U at site 7 to destroy this similarity in seed sequences of anti-146b-3p (Figure 1b). Additional 5′CCC and 3′TTTTTA flanking sequences (both adopted from the pSUPER RNAi System^TM^ manual, OligoEngine, Inc.) were added after attaching cohesive ends compatible with *Bam*H1 and *Eco*R1 sites, which were used for cloning inserts into the plvx-shRNA2 vector (Figure 1c). Finally, we annealed the two synthesized template DNA oligos and inserted the DNA fragment into the plvx-shRNA2 vector (Figure 1c), using sequencing to confirm correct insertion.

### Successful overexpression of hsa-miR-146b-3p from the plvx-hs-146b-3p plasmid

**Figure 2:**
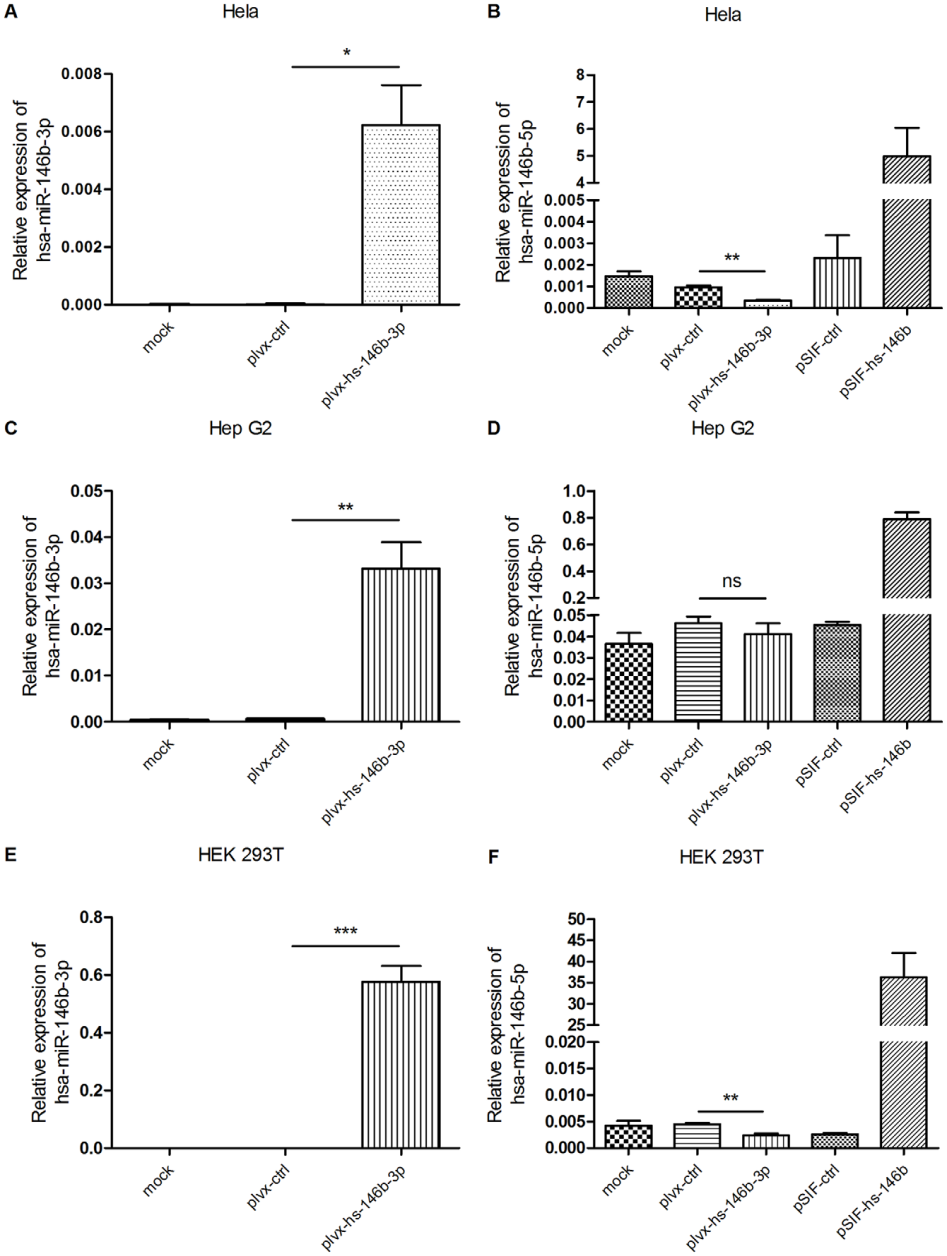
Successful overexpression of hsa-miR-146b-3p by plvx-hs-146b-3p with no detectable increase of hsa-miR-146b-5p. HeLa cells were transfected with the indicated plasmids (400 ng per well) and RNA was collected and extracted 24 h later. The expression of hsa-miR-146b-3p (A) and hsa-miR-146b-5p (B) was detected using qRT–PCR. The same experiments were done in Hep G2 cells (C) for hsa-miR-146b-3p; (D) for hsa-miR-146b-5p and HEK 293T cells (E) for hsa-miR-146b-3p; (F) for hsa-miR-146b-5p. Each graph shows the mean of three independent experiments that measured the relative expression levels (2^−deltaCT^) of the two miRNAs to the reference gene RNU48. Error bars represent SEMs. * means p value ≤0.05; ** means p value ≤0.01; *** means p value ≤0.001; ns means no significance.

To verify our overexpression strategy and ensure that plvx-hs-146b-3p could indeed overexpress hsa-miR-146b-3p without affecting the expression level of hsa-miR-146b-5p, we transfected HeLa cells with plvx-hs-146b-3p plasmids. Once 24 h had passed after transfection, RNA was collected and the expression of the two miRNAs was measured by real-time PCR using the TaqMan® MicroRNA Assay. As shown in Figure 2a, there was a high level expression of hsa-miR-146b-3p compared to levels following mock transfection or transfection with an empty control vector (plvx-ctrl). For the examination of hsa-miR-146b-5p in plvx-hs-146b-3p transfected cells, we used another plasmid (referred as pSIF-hs-146b), which had the ability to overexpress hsa-miR-146b-5p, as a positive overexpression control for hsa-miR-146b-5p. As expected, pSIF-hs-146b achieved a high level of hsa-miR-146b-5p expression compared with either mock transfection or transfection with pSIF-ctrl, the empty control vector for pSIF-hs-146b. We could not detect any overexpressed hsa-miR-146b-5p in the sample that was transfected with plvx-hs-146b-3p (Figure 2b). In addition, we verified these results using another two cell lines (Figure 2c–2f).

**Figure 3:**
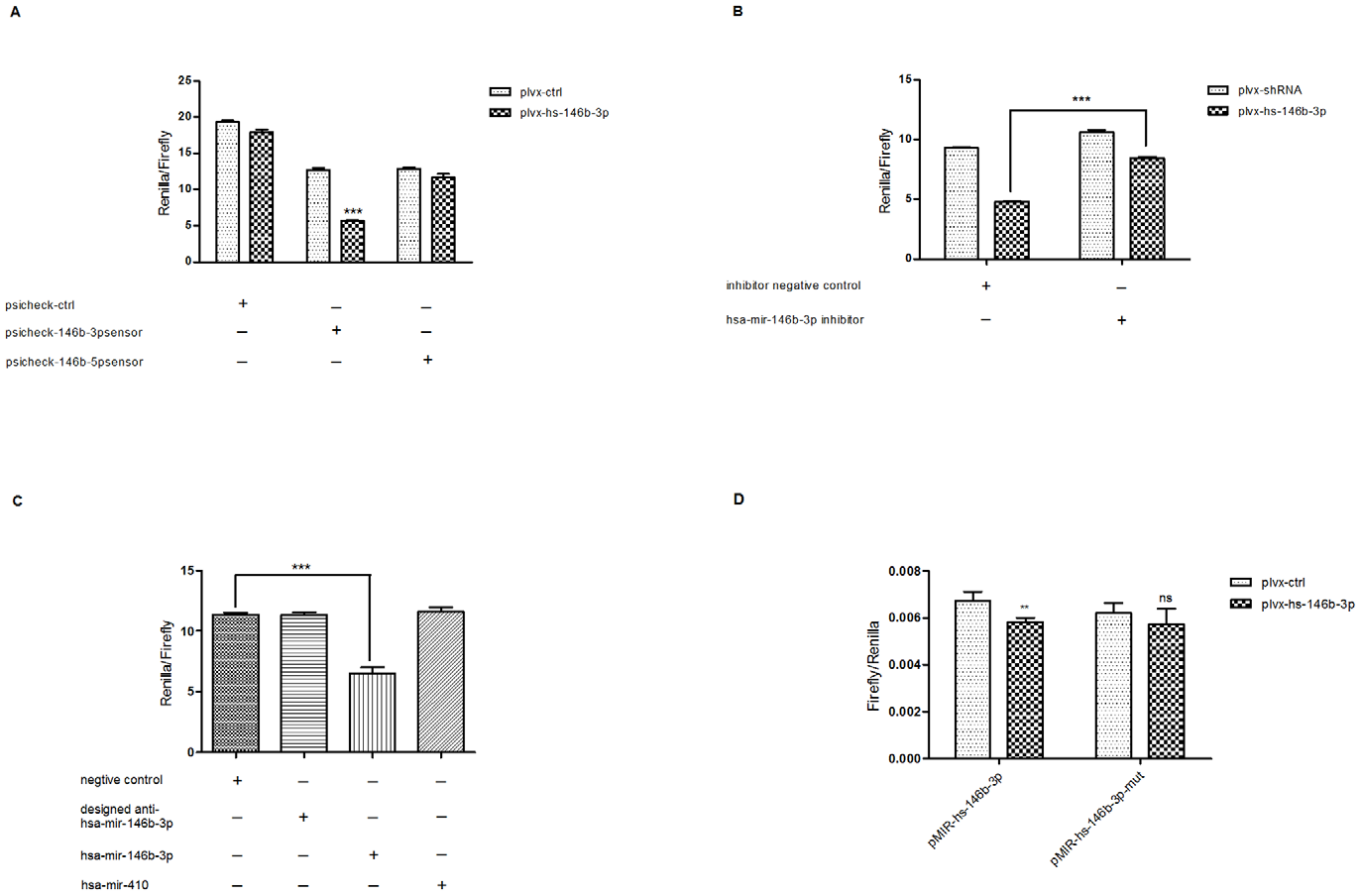
plvx-hs-146b-3p overexpressed functional hsa-miR-146b-3p with no detectable activity change of hsa-miR-146b-5p. (A) Transcription products from plvx-hs-146b-3p had hsa-miR-146b-3p activities, without changing the activity of hsa-miR-146b-5p. HeLa cells were transfected with psicheck-ctrl (20 ng per well), psicheck-146b-3psensor (20 ng per well), or psicheck-146b-5psensor (20 ng per well), in combination with either plvx-ctrl (100 ng per well) or plvx-hs-146b-3p (100 ng per well), as indicated in the graph. (B) The repression of psicheck-146b-3psensor activity could be rescued using hsa-miR-146b-3p inhibitors. HeLa cells were transfected with psicheck-146b-3psensor (20 ng per well) in combination of plvx-ctrl (100 ng per well) or plvx-hs-146b-3p (100 ng per well) under the presentation of hsa-miR-146b-3p inhibitors (100 nM) or inhibitor negative controls (100 nM), as indicated in the graph. (C) Designed modified complementary sequences (designed anti-hsa-miR-146b-3p) could not repress psicheck-146b-3psensor activity as effectively as hsa-miR-146b-3p. HeLa cells were transfected with psicheck-146b-3psensor (20 ng per well) in combination with single stranded RNA mimics of negative control sequences (100 nM), designed anti-hsa-miR-146b-3p (100 nM), hsa-miR-146b-3p (100 nM), and hsa-miR-410 (100 nM), as indicated in the graph. The miRNA hsa-miR-410, which had no ability to repress psicheck-146b-3psensor activity, was used as a negative control miRNA. (D) The miRNA hsa-miR-146b-3p overexpressed by plvx-hs-146b-3p could repress the activity of bulged sensor of hsa-miR-146b-3p (pMIR-hs-146b-3p) and this repression was abolished by mutation of the seed sequences of the bulged sensor. HeLa cells were transfected with pMIR-hs-146b-3p (2 ng per well) or pMIR-hs-146b-3p-mut (2 ng per well), together with plvx-ctrl (400 ng per well) or plvx-hs-146b-3p (400 ng per well), as indicated in the graph. Renilla luciferase vector (5 ng per well) was delivered simultaneously as a transfection control. For all assays, protein was collected 24 h after transfection. Luciferase activity was quantified and expressed as relative luciferase activity. The data represent one of at least three independent experiments, each of which involved four replicates. Error bars represent SEMs. ** means p value ≤0.01; *** means p value ≤0.001; ns means no significance.

Meanwhile, we noticed that hsa-miR-146b-5p was reduced in both Hela and HEK 293T cells transfected with plvx-hs-146b-3p. We suspected that this was due to an inaccurate quantification of hsa-miR-146b-5p, because there was no significant change of the activity of hsa-miR-146b-5p detected by luciferase sensor experiment (Figure 3a, Figure S3a, Figure S3b).

To test if the overexpressed hsa-miR-146b-3p or anti-146-3p could affect the accuracy of the quantification of hsa-miR-146b-5p, we used mixed RNA samples containing different amount of synthetic single stranded hsa-miR-146b-3p (or anti-146b-3p) RNA (100 ng, 10 ng, 1 ng, 0.1 ng, 0.01 ng, 0.001 ng, 0.0001 ng in each sample) and equal amount of hsa-miR-146b-5p containing RNA (20 ng in each sample) for the quantification of hsa-miR-146b-5p. While the synthetic single stranded anti-146b-3p did not affect the quantification of hsa-miR-146b-5p, synthetic single stranded hsa-miR-146b-3p reduced the detected level of hsa-miR-146b-5p in a dose dependent manner (Figure S1a). The more hsa-miR-146b-3p was added, the less hsa-miR-146b-5p was detected (the higher Ct value was observed). We then did the reverse transcription of synthetic single stranded hsa-miR-146b-3p (100 ng, 10 ng, 1ng, 0.1 ng, 0.01 ng in each reaction) and hsa-miR-146b-5p containing RNA (20 ng in each reaction) separately, and combined the products of each hsa-miR-146b-3p sample (100 ng, 10 ng, 1ng, 0.1 ng, 0.01 ng in each reaction) with that of each hsa-miR-146b-5p containing RNA sample (20 ng in each reaction) and used the mixed samples for subsequent hsa-miR-146b-5p detection. This time, we did not observed any dose dependent effects on the Ct values of hsa-miR-146b-5p (Figure S1b). Thus, we confirmed that the inaccurate quantification was resulted from the deficient reverse transcription of hsa-miR-146b-5p. Finally, we used miScript 2 RT kit (from QIAGEN), which applied a different principle for reverse transcription of miRNAs, to do reverse transcription and quantified hsa-miR-146b-5p by SYBR-green based method using the same sample in Figure 2f (which was mock, plvx-ctrl, and plvx-hs-146b-3p). As shown in Figure S1c, although it was slightly lower than the control samples (mock and plvx-ctrl), the significant reduced expression of hsa-miR-146b-5p was not observed. So, we proposed that the reduction of hsa-miR-146b-5p was due to the effect of abundant exogenous hsa-miR-146b-3p on the efficiency of hsa-miR-146b-5p's reverse transcription when using Taqman assay and it was possibly caused by the paring of the complimentary sequences between hsa-miR-146b-3p and hsa-miR-146b-5p (Figure S1d), which might affect efficient primer binding.

Considering that different cell lines have different endogenous levels of hsa-miR-146b-5p and transfection efficiency of different cell lines was varied, the ratio of hsa-miR-146b-3p to hsa-miR-146b-5p in the RNA samples from different cell lines was not the same and the extent of this effect was varied too. Therefore, the observed reduction of hsa-miR-146b-5p showed a cell type specific phenomenon (Hela and HEK 293T cells were more easily transfected and contained much lower level of hsa-miR-146b-5p than Hep G2 cells).

Collectively, these data demonstrated that our plvx-hs-146b-3p plasmid can overexpress hsa-miR-146b-3p without altering the expression of hsa-miR-146b-5p. Moreover, because the stem–loop structure of the reverse transcription primers used in the TaqMan® MicroRNA Assay provided specificity for only the mature miRNA with its entire 3′ end [42], we could confidently conclude that the over-expressed hsa-miR-146b-3p in our experiments was identical to the mature form in miRBase, which represented the most abundant sequence of the miRNA [39].

### The overexpressed hsa-miR-146b-3p by plvx-hs-146b-3p functioned as a miRNA without detectable change of the activity of hsa-miR-146b-5p

To analyze whether the miRNA overexpressed by our plvx-hs-146b-3p plasmid was functional and further exclude the possible existence of the side effects associated with the change of the expression of hsa-miR-146b-5p, we first constructed sensor plasmids for both hsa-miR-146b-3p (referred to as psicheck-hs-146b-3p) and hsa-miR-146b-5p (referred to as psicheck-hs-146b-5p). These contain two perfect binding sites in the 3′UTR region of Renilla luciferase reporter gene, which would provide good sensitivity to monitor miRNA activities previously described [29]. We confirmed that these sensor plasmids could indeed sense the activities of their corresponding miRNAs by co-transfecting them with synthetic miRNA mimics for hsa-miR-146b-3p and hsa-miR-146b-5p, respectively (Figure S2a and Figure S2b). More importantly, psicheck-hs-146b-5p could not be affected by hsa-miR-146b-3p mimics (Figure S2b) and another unrelated miRNA hsa-miR-218 could not inhibit Renilla luciferase activity expressed from psicheck-hs-146b-3p (Figure S2a). This indicated that the decreased luciferase signal was specific for the activity of the corresponding miRNA.

We then transfected HeLa cells with either psicheck-hs-146b-3p or psicheck-hs-146b-5p in combination with plvx-hs-146b-3p or plvx-ctrl, respectively. Consistent with previous miRNA expression data, plvx-hs-146b-3p could only repress the Renilla luciferase activity of psicheck-hs-146b-3p with no inhibition of activity arising from psicheck-hs-146b-5p (Figure 3a). We further eliminated the possibility that plvx-hs-146b-3p could repress activity of the Renilla luciferase reporter gene independent of the 3′UTR by transfecting plvx-hs-146b-3p together with an empty psicheck vector, named psicheck-ctrl (Figure 3a). To exclude a cell type specific effect, we did the same experiments in HEK 293T and Hep G2 cells and got the same results (Figure S3).

To confirm that the repression was indeed mediated by overexpressed hsa-miR-146b-3p and not through a nonspecific effect caused by transfection with an exogenous plasmid, we transfected inhibitors of hsa-miR-146b-3p together with plvx-hs-146b-3p to block the activity of hsa-miR-146b-3p. As expected, the repression was almost fully rescued by the inhibition of hsa-miR-146b-3p (Figure 3b).

Considering that the sequences located on either side of the stem of an shRNA had the capability to mature and might be functional [43], [44], we needed to exclude the possibility that the repression of plvx-hs-146b-3p sensor's activity may be caused by non-specific effects of the other strand. To overexpress the two sequences from the stem of the designed shRNA, we synthesized single-strand RNAs with the same sequences of both hsa-miR-146b-3p and its designed counterpart. We then transfected them into HeLa cells separately, both with psicheck-hs-146b-3p. As expected, only single-stand hsa-miR-146b-3p could repress the activity of Renilla luciferase from psicheck-hs-146b-3p (Figure 3c).

MicroRNAs usually function by incomplete complementary pairing to their target region, in which process perfect seed pairing is essential [41]. Therefore, to further validate that the miRNA produced by our plasmid could function as typical miRNAs other than only as siRNAs, we constructed another sensor plasmid (pMIR-hs-146b-3p) with two tandem target sites containing central bulges to mimic the traditional miRNA targets as previous reported [29]. We then mutated the seed region of the bulged target site to generate the plasmid pMIR-hs-146b-3p-mut. Having confirmed that pMIR-hs-146b-3p could indeed sense the activity of hsa-miR-146b-3p and the mutation in pMIR-hs-146b-3p-mut could abolish this effect by using miRNA mimics of hsa-miR-146b-3p (Figure S4), we then tested the repression abilities of our plvx-hs-146b-3p plasmids by these two sensor plasmids. As shown in Figure 3d, plvx-hs-146b-3p repressed bulged sensor activity and this effect was abolished by mutation of its seed region. This indicated a miRNA-like function of the overexpressed miRNA.

Taken together, we demonstrated that our prototype plasmid plvx-hs-146b-3p could specifically overexpress hsa-miR-146b-3p, which could function as effectively as natural miRNAs without any activity change of hsa-miR-146b-5p.

### Overexpression of other miRNAs by our strategy

**Figure 4:**
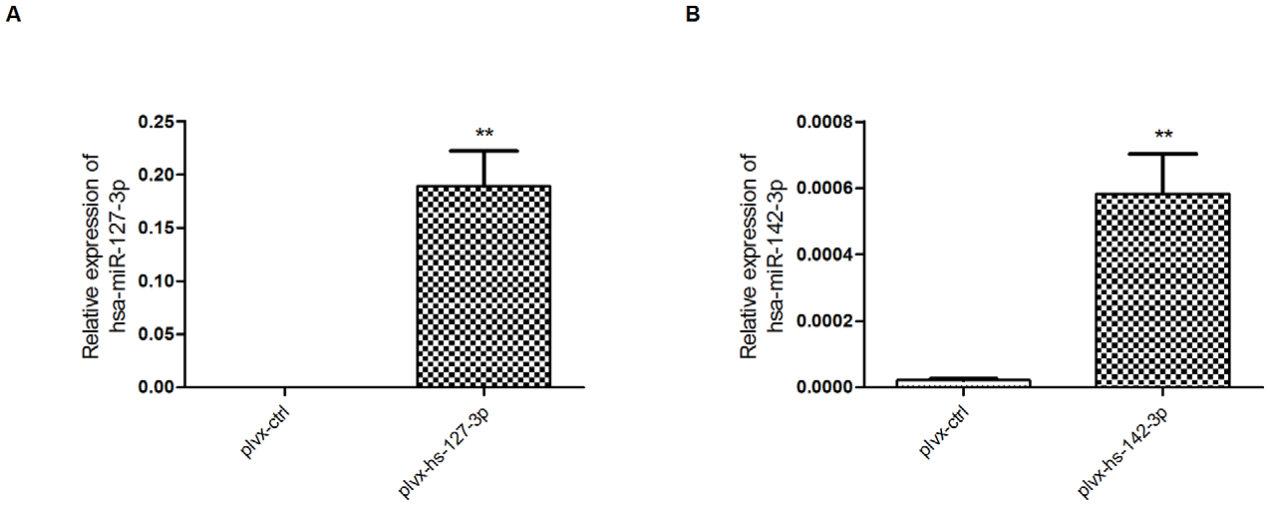
Successful overexpression of hsa-miR-127-3p and hsa-miR-142-3p. HeLa cells were transfected with the indicated plasmids (400 ng per well) and RNA was collected and extracted 24 h later. Levels of expression of hsa-miR-127-3p (A) and hsa-miR-142-3p (B) were detected using qRT–PCR. Each graph shows the representative result of at least three independent experiments. Relative expression levels (2^−deltaCT^), to the reference gene RNU48, were plotted. Error bars represent SEMs. ** means p value ≤0.01.

In order to see if our strategy could overexpress other miRNAs, we synthesized customized sequences for hsa-miR-127-3p and hsa-miR-142-3p, which were selected to work with because we already had their cognate detection probes. The corresponding overexpression plasmids we constructed were named plvx-hs-127-3p and plvx-hs-142-3p, respectively. As shown in Figure 4, both miRNAs were successfully overexpressed from their corresponding plasmids in HeLa cells. This indicates that our strategy can be used to overexpress different miRNAs, including highly expressed miRNA species such as hsa-miR-127-3p.

## Discussion

MicroRNAs, which make a critical contribution to the regulation of gene expression circuits, participate in many physiological and pathological processes by fine-tuning signaling events at the molecular and cellular levels [1], [8]. Intensive effort has been devoted to exploring their functions. Any single miRNA precursor potentially produces two mature miRNAs [12]. However, the function of the less expressed form, the miRNA* species, has not been studied as intensively as that of its more highly expressed miRNA counterpart. Recently, many expression data sets and large-scale small RNA sequencing efforts revealed a tissue- or cell-type-dependent accumulation of miRNA* species [45]. Our own unpublished data also showed that different physiological and environmental stimuli can change the profile of mature miRNAs in certain cell types, including miRNA* species. Additionally, recent work demonstrated that miRNA* species can have important effects on vertebrate regulatory networks, suggesting the importance of studies that explore their functions and could potentially contribute to better understanding of disease states [29], [30], [31].

Synthetic miRNA mimics are often used to introduce specific miRNAs into target cells. However, transient overexpression cannot provide sufficiently high long-term expression and *in vivo* delivery is still difficult. Vector-based overexpression methods, especially those involving viral vectors, provide an opportunity to overcome this problem. But, because of the nature of the biogenesis of miRNAs, cells tend to express miRNAs other than the sparsely expressed star forms. This constitutes the main theoretical and practical obstacle for using traditional cloning methods to construct miRNA overexpression vectors for expressing miRNA* species.

By mimicking the design of shRNAs, we developed a strategy to overexpress miRNA* species without detectable increase of their highly expressed counterparts. This enabled us to investigate the function of miRNA* species without side effects introduced by expression of their miRNA counterparts. As mentioned above, there were many unpaired bases in the predicted secondary structures of the miRNA precursors [39], [40], and the two miRNAs from the same precursor usually have 3′ overhang bases in their own sequences. Therefore, the complete complementary sequences of the miRNA* species could not be the same as the original miRNA from the other strand. This suggested to us the potential value of manipulating the complementary sequences of the miRNA* species in order to eliminate any activity of its counterpart miRNA. Because both strands from the shRNA had the possibility to function [43], [44], two principles warrant particular mention. The first is the importance of changing several sequences within the seed region in the complementary sequences that still had the same bases as the original miRNA to ensure that its activity is eliminated. The second is the importance of checking the potential siRNA effects of the complementary sequences, and excluding related side effects by mutating some of the nucleotides. Additionally, different mutation strategies could be used to make anti-miRNA* strands and construct different plasmids. By checking if the phenomenon is the same when using two different plasmids, one can make the decision about if their phenomenon is really associated with miRNA* or not.

Another consideration concerns whether levels of expression level are sufficiently high. Because of the differences between individual miRNAs, not all designed stem-loop structures enable very high levels of expression. We thought that this might arise as a consequence of the different efficiency with which mature sequences are incorporated into the RISC complex. Previous studies showed that the strand with its 5′ end less tightly paired to the complementary sequences has lower internal stability, which makes it bind the RISC complex more efficiently than the other strand [13], [14]. Thus, we suggested that additional mutation could be used to modify the 3′ end of the complementary sequences to increase levels of expression. In summary, we could express specific miRNA* species by an designed shRNA with miRNA* sequences in the anti-sense strand and manipulated complementary sequences in the sense strand. Using this strategy, combined with different vector systems, researchers should be able to easily design and make their own overexpression plasmids without the understanding aspects of the structure and processing principles of miRNA precursors that remain to be fully elucidated.

Vector-based miRNA expression remains a convenient tool for long-term overexpression experiments, especially given its capability to stable express specific miRNAs in live cells. Moreover, the use of viral vectors solved the problem of difficult transfection and different viral vectors were widely used in *in vivo* studies. Therefore, by combining different vector systems, our strategy should enable researchers to explore the function of any miRNA* or even miRNA species *in vitro* or *in vivo* without interference from related molecules normally generated from the same pre-miRNA. Moreover, considering the involvement of miRNAs in many diseases, including cancer [46] and autoimmune diseases [47], the capacity to specifically alter the abundance of certain miRNAs potentially provides a new strategy for treating human diseases [48]. And our strategy provides such a tool for overexpressing one mature miRNA in patients without unwanted side effects arising from the simultaneous expression of the other miRNA encoded by the same precursor.

Collectively, our work presents a new way to overexpress functional miRNA* species, which has been challenging to achieve using traditional methods. Further experiments demonstrated that the strategy can be applied to different miRNA* species or even miRNA species. By taking advantage of different vector expression systems, our approach should find useful applications both for experimental research or therapeutic applications.

## Supporting Information

Figure S1 **Highly expressed hsa-miR-146b-3p affected the quantification of endogenous hsa-miR-146b-5p using Taqman method.** (A) Different amount of synthetic single stranded hsa-miR-146b-3p RNA (indicated on the X axis) was added to each equal amount of hsa-miR-146b-5p containing RNA (20 ng), and then the RNA mixtures were used for quantification using Taqman assay for hsa-miR-146b-5p. Ct values were shown on the Y axis. (B) Different amount of synthetic single stranded hsa-miR-146b-3p RNA (indicated on the X axis) and five pieces of equal amount of hsa-miR-146b-5p containing RNA (20 ng) were reverse transcribed using Taqman assay for hsa-miR-146b-5p. The products from each synthetic single stranded hsa-miR-146b-3p RNA sample were put together with that from each equal amount of hsa-miR-146b-5p containing RNA, and then the cDNA mixtures were used for subsequent quantification using Taqman probes for hsa-miR-146b-5p. Ct values were shown on the Y axis. (C) The same RNA samples used in Figure 2f (named mock, plvx-ctrl, and plvx-hs-146b-3p) were transcribed using miScript 2 RT kit (QIAGEN), and hsa-miR-146b-5p was detected by SYBR green method. (D) shows the complimentary sequences between hsa-miR-146b-3p and hsa-miR-146b-5p.
(TIF)

Figure S2 **Validation of the sensitivity and specificity of psicheck-146b-3psensor and psicheck-146b-5psensor.** HeLa cells were transfected with (A) psicheck-146b-3psensor (20 ng per well) with either of the miRNA mimics hsa-miR-146b-3p or hsa-miR-218 (100 nM), and (B) psicheck-146b-5psensor (20 ng per well) with either of the miRNA mimics hsa-miR-146b-3p or hsa-miR-146b-5p (100 nM), as indicated in the graph. After 24 h incubation, the protein was collected and luciferase activity was quantified and expressed as the relative luciferase activity. The data represent at one of at least three independent experiments, each of which involved four replicates. Error bars represent SEMs. *** means p value ≤0.001.
(TIF)

Figure S3 **plvx-hs-146b-3p overexpressed functional hsa-miR-146b-3p with no detectable activity change of hsa-miR-146b-5p in HEK 293T and Hep G2 cells.** Transcription products from plvx-hs-146b-3p had hsa-miR-146b-3p activities, without changing the activity of hsa-miR-146b-5p. HEK 293T cells (A) or Hep G2 cells (B) were transfected with psicheck-ctrl (20 ng per well), psicheck-146b-3psensor (20 ng per well), or psicheck-146b-5psensor (20 ng per well), in combination with either plvx-ctrl (100 ng per well) or plvx-hs-146b-3p (100 ng per well), as indicated in the graph. For all assays, protein was collected 24 h after transfection. Luciferase activity was quantified and expressed as relative luciferase activity. The data represent one of at least three independent experiments, each of which involved four replicates. Error bars represent SEMs. *** means p value ≤0.001.
(TIF)

Figure S4 **Validation of the sensitivity and specificity of pMIR-hs-146b-3p and pMIR-hs-146b-3p-mut.** HeLa cells were transfected with pMIR-hs-146b-3p (2 ng per well) (A) or pMIR-hs-146b-3p-mut (2 ng per well) (B) in combination of miRNA mimics (100 nM) of hsa-miR-146b-3p or negative control sequences respectively as indicated in the graph. Renilla luciferase vectors were simultaneously transfected (5 ng per well) as a transfection control. After 24 h incubation, the protein was collected and luciferase activity was quantified and expressed as the relative luciferase activity. The data represent one of at least three independent experiments, each of which involved four replicates. Error bars represent SEMs. *** means p value ≤0.001; ns means no significance.
(TIF)
