# Supplementary figures and images for: A Novel Vector-Based Method for Exclusive Overexpression of Star-Form MicroRNAs
Source: PLoS One. 2012 Jul 19;7(7):e41504. doi: 10.1371/journal.pone.0041504 (PMC3400617; doi:10.1371/journal.pone.0041504)

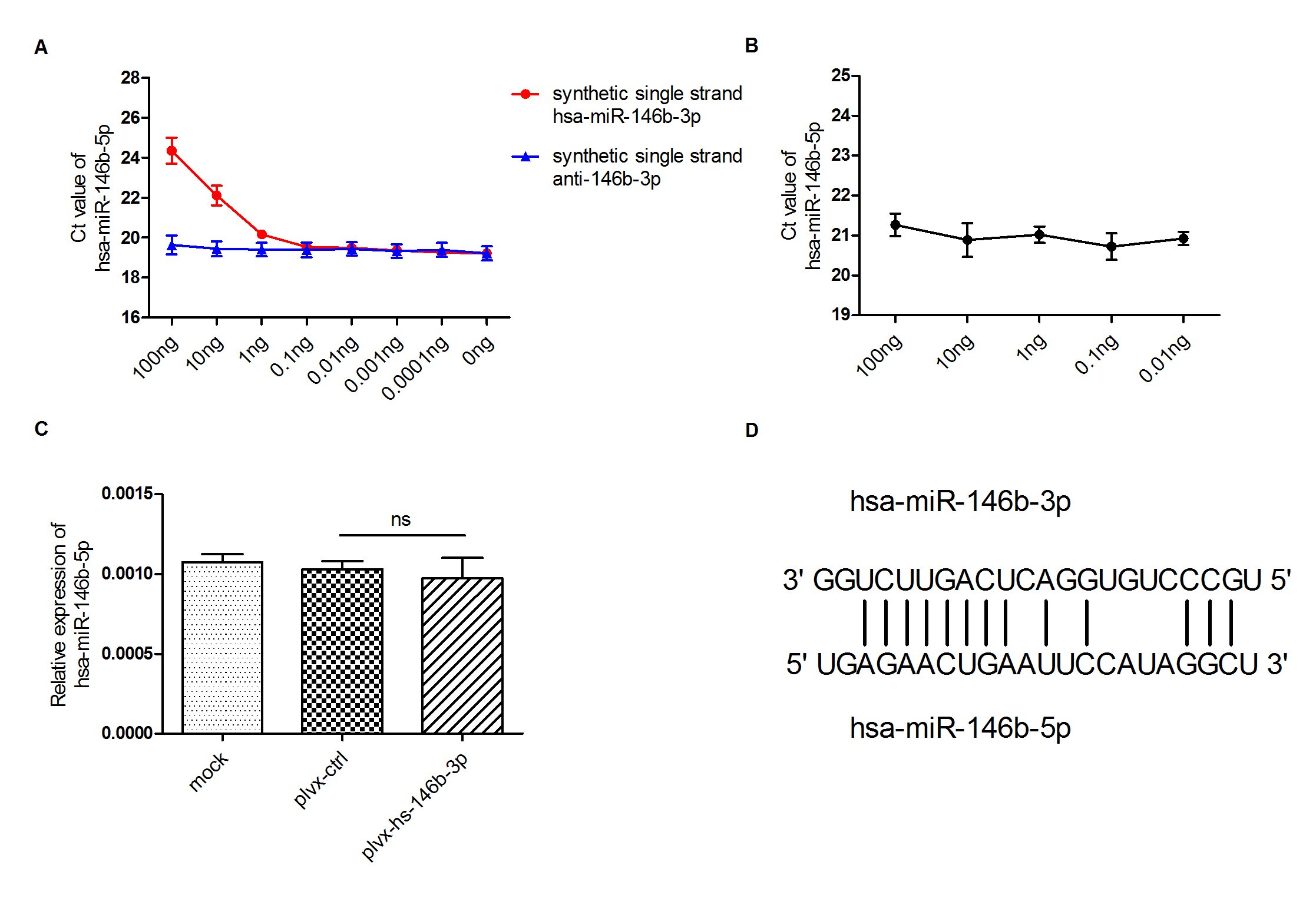

Supplement: Figure S1 — Highly expressed hsa-miR-146b-3p affected the quantification of endogenous hsa-miR-146b-5p using Taqman method. (A) Different amount of synthetic single stranded hsa-miR-146b-3p RNA (indicated on the X axis) was added to each equal amount of hsa-miR-146b-5p containing RNA (20 ng), and then the RNA mixtures were used for quantification using Taqman assay for hsa-miR-146b-5p. Ct values were shown on the Y axis. (B) Different amount of synthetic single stranded hsa-miR-146b-3p RNA (indicated on the X axis) and five pieces of equal amount of hsa-miR-146b-5p containing RNA (20 ng) were reverse transcribed using Taqman assay for hsa-miR-146b-5p. The products from each synthetic single stranded hsa-miR-146b-3p RNA sample were put together with that from each equal amount of hsa-miR-146b-5p containing RNA, and then the cDNA mixtures were used for subsequent quantification using Taqman probes for hsa-miR-146b-5p. Ct values were shown on the Y axis. (C) The same RNA samples used in Figure 2f (named mock, plvx-ctrl, and plvx-hs-146b-3p) were transcribed using miScript 2 RT kit (QIAGEN), and hsa-miR-146b-5p was detected by SYBR green method. (D) shows the complimentary sequences between hsa-miR-146b-3p and hsa-miR-146b-5p. (TIF) [file pone.0041504.s001.tif]

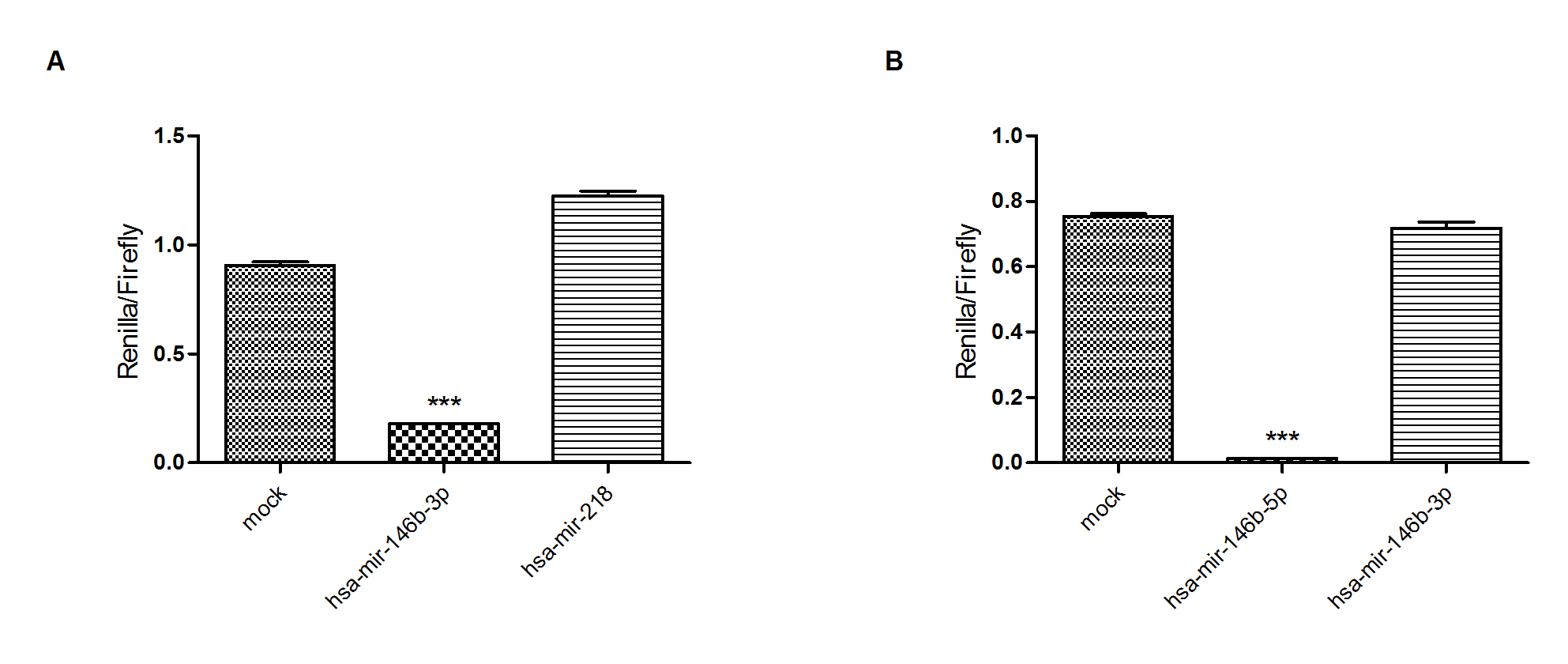

Supplement: Figure S2 — Validation of the sensitivity and specificity of psicheck-146b-3psensor and psicheck-146b-5psensor. HeLa cells were transfected with (A) psicheck-146b-3psensor (20 ng per well) with either of the miRNA mimics hsa-miR-146b-3p or hsa-miR-218 (100 nM), and (B) psicheck-146b-5psensor (20 ng per well) with either of the miRNA mimics hsa-miR-146b-3p or hsa-miR-146b-5p (100 nM), as indicated in the graph. After 24 h incubation, the protein was collected and luciferase activity was quantified and expressed as the relative luciferase activity. The data represent at one of at least three independent experiments, each of which involved four replicates. Error bars represent SEMs. *** means p value ≤0.001. (TIF) [file pone.0041504.s002.tif]

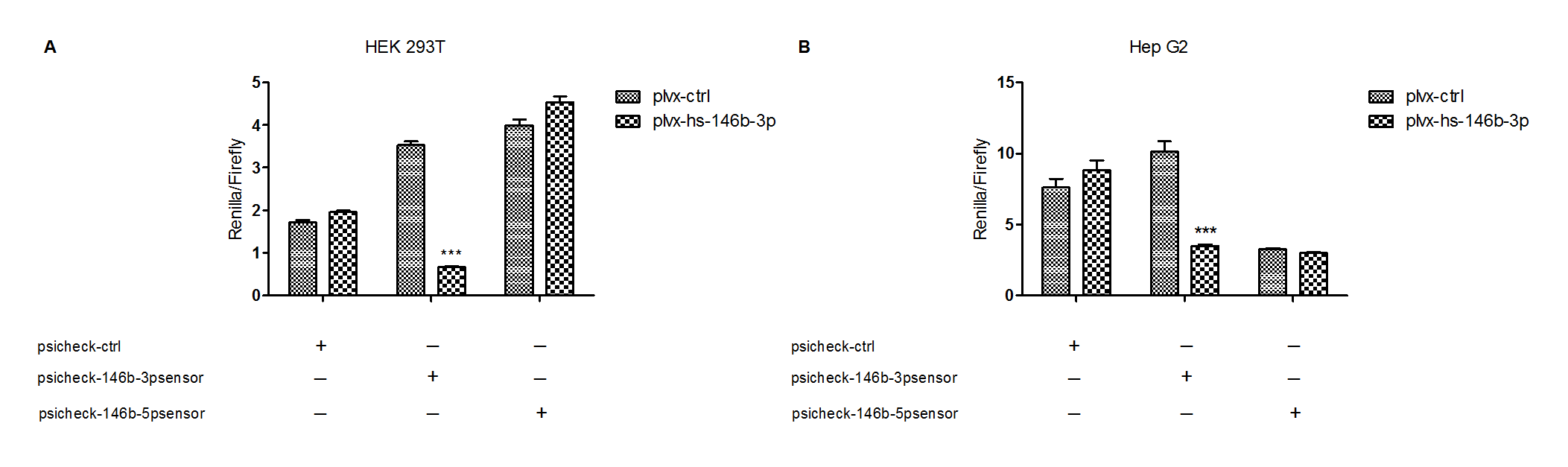

Supplement: Figure S3 — plvx-hs-146b-3p overexpressed functional hsa-miR-146b-3p with no detectable activity change of hsa-miR-146b-5p in HEK 293T and Hep G2 cells. Transcription products from plvx-hs-146b-3p had hsa-miR-146b-3p activities, without changing the activity of hsa-miR-146b-5p. HEK 293T cells (A) or Hep G2 cells (B) were transfected with psicheck-ctrl (20 ng per well), psicheck-146b-3psensor (20 ng per well), or psicheck-146b-5psensor (20 ng per well), in combination with either plvx-ctrl (100 ng per well) or plvx-hs-146b-3p (100 ng per well), as indicated in the graph. For all assays, protein was collected 24 h after transfection. Luciferase activity was quantified and expressed as relative luciferase activity. The data represent one of at least three independent experiments, each of which involved four replicates. Error bars represent SEMs. *** means p value ≤0.001. (TIF) [file pone.0041504.s003.tif]

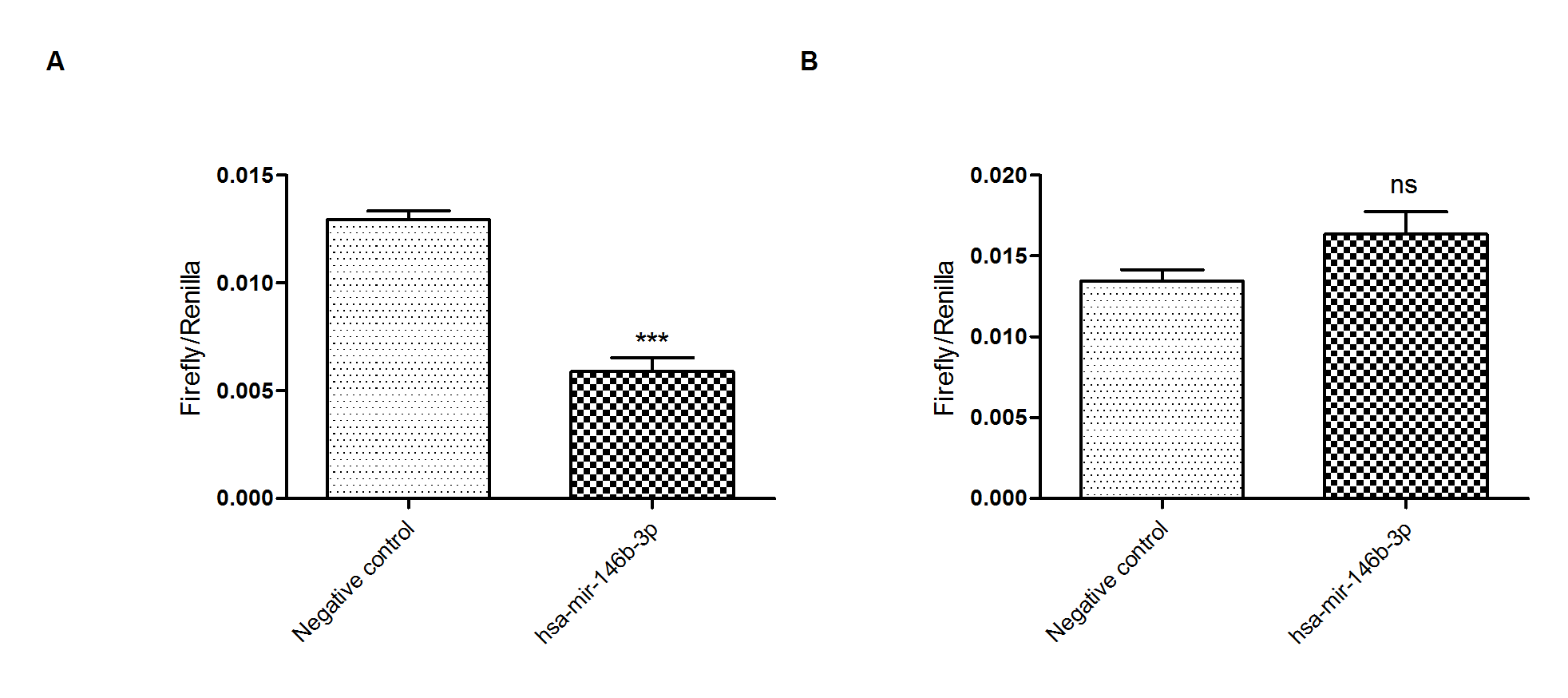

Supplement: Figure S4 — Validation of the sensitivity and specificity of pMIR-hs-146b-3p and pMIR-hs-146b-3p-mut. HeLa cells were transfected with pMIR-hs-146b-3p (2 ng per well) (A) or pMIR-hs-146b-3p-mut (2 ng per well) (B) in combination of miRNA mimics (100 nM) of hsa-miR-146b-3p or negative control sequences respectively as indicated in the graph. Renilla luciferase vectors were simultaneously transfected (5 ng per well) as a transfection control. After 24 h incubation, the protein was collected and luciferase activity was quantified and expressed as the relative luciferase activity. The data represent one of at least three independent experiments, each of which involved four replicates. Error bars represent SEMs. *** means p value ≤0.001; ns means no significance. (TIF) [file pone.0041504.s004.tif]
